# Evolution of the MAGUK protein gene family in premetazoan lineages

**DOI:** 10.1186/1471-2148-10-93

**Published:** 2010-04-01

**Authors:** Alex de Mendoza, Hiroshi Suga, Iñaki Ruiz-Trillo

**Affiliations:** 1 Departament de Genètica & Institut de Recerca en Biodiversitat (Irbio), Universitat de Barcelona, Barcelona, Spain; 2 Institució Catalana per a la Recerca i Estudis Avançats (ICREA); Parc Científic de Barcelona, Baldiri Reixach 15, 08028 Barcelona, Spain

## Abstract

**Background:**

Cell-to-cell communication is a key process in multicellular organisms. In multicellular animals, scaffolding proteins belonging to the family of membrane-associated guanylate kinases (MAGUK) are involved in the regulation and formation of cell junctions. These MAGUK proteins were believed to be exclusive to Metazoa. However, a MAGUK gene was recently identified in an EST survey of *Capsaspora owczarzaki*, an unicellular organism that branches off near the metazoan clade. To further investigate the evolutionary history of MAGUK, we have undertook a broader search for this gene family using available genomic sequences of different opisthokont taxa.

**Results:**

Our survey and phylogenetic analyses show that MAGUK proteins are present not only in Metazoa, but also in the choanoflagellate *Monosiga brevicollis *and in the protist *Capsaspora owczarzaki*. However, MAGUKs are absent from fungi, amoebozoans or any other eukaryote. The repertoire of MAGUKs in Placozoa and eumetazoan taxa (Cnidaria + Bilateria) is quite similar, except for one class that is missing in *Trichoplax*, while Porifera have a simpler MAGUK repertoire. However, Vertebrata have undergone several independent duplications and exhibit two exclusive MAGUK classes. Three different MAGUK types are found in both *M. brevicollis *and *C. owczarzaki: DLG, MPP and MAGI*. Furthermore, *M. brevicollis *has suffered a lineage-specific diversification.

**Conclusions:**

The diversification of the MAGUK protein gene family occurred, most probably, prior to the divergence between Metazoa+choanoflagellates and the *Capsaspora*+*Ministeria *clade. A MAGI-like, a DLG-like, and a MPP-like ancestral genes were already present in the unicellular ancestor of Metazoa, and new gene members have been incorporated through metazoan evolution within two major periods, one before the sponge-eumetazoan split and another within the vertebrate lineage. Moreover, choanoflagellates have suffered an independent MAGUK diversification. This study highlights the importance of generating enough genome data from the broadest possible taxonomic sampling, in order to fully understand the evolutionary history of major protein gene families.

## Background

The emergence of multicellular animals from their protist ancestors brought evolutionary novelties together with some significant genetic challenges. For example, it is believed that the genes involved in cell-cell communication, cell adhesion and cell differentiation probably arose before or concomitantly with the origins of multicellularity [1]. One of the protein families involved in cell-to-cell communication in Metazoa is the family of scaffolding proteins known as membrane-associated guanylate kinases (MAGUKs), which organize protein complexes at cell or synaptic junctions (for a review see [2]). The MAGUKs have a wide variety of biological roles, such as regulating cell polarity [3], connecting transmembrane proteins (or actin filaments) with the cytoskeleton in tight junctions [4-6], and regulating synapse formation and plasticity [7-9]. Therefore, MAGUKs are of critical importance to the development of multicellular animals.

**Figure 1:**
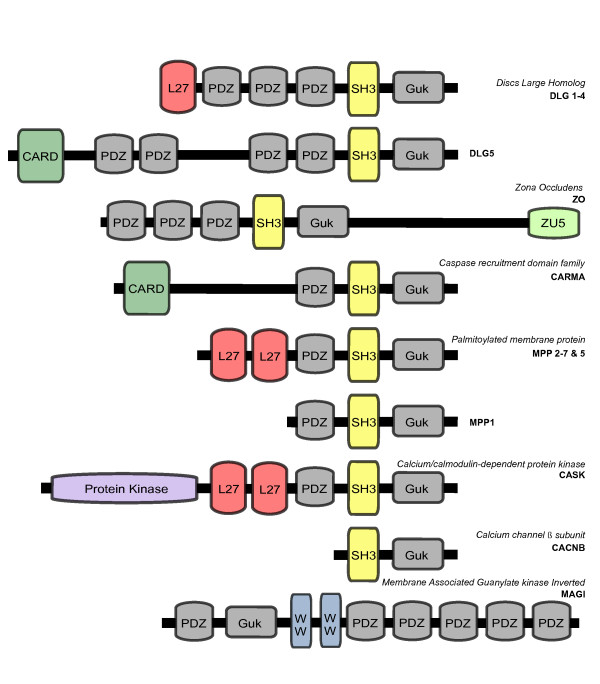
**Domain architectures of the different MAGUK classes**. Domain architectures of the different MAGUK classes. Only the canonical form is shown for each class.

The MAGUK family have been divided into different classes or groups, according to phylogenetic position and protein domain architecture (see for example [2] and [10], and Figure 1 for our own MAGUK classification). These MAGUKs classes are known as calcium/calmodulin-dependent proteins kinase (CASK), palmitoylated membrane protein (MPP), zona occludens (ZO), caspase recruitment domain family (CARMA), Disc Large Homolog (DLG), Calcium channel β subunit (CACNB), and membrane-associated guanylate kinase with an inverted repeat (MAGI). All classes contain the following: one or several PDZ domains (except CACNB), a catalytically inactive guanylate kinase (GUK) domain with homology to yeast guanylate kinase and a Src Homology 3 (SH3) domain (except for MAGI) (Figure 1). Members of the MAGI class, on the other hand, have two WW (conserved Trp residue) domains instead of the SH3 domain. These WW domains are situated downstream of the GUK domain (unlike all other MAGUKs). All of these modular motifs in MAGUK mediate protein-protein interactions.

The MAGUK protein gene family had been considered to be exclusive to Metazoa [10,11] and, hence, a key gene family for determining metazoan origins. However, a recent EST survey showed that an homolog of MAGI is present in the protist *C. owczarzaki *[12], which seems to be the sister-group to the Choanoflagellata+Metazoa clade. This finding led to new questions, such as whether other MAGUKs were already present in the ancestor of Metazoa, the time of divergence of this gene family, and whether choanoflagellates also had MAGUK homologs. To answer these questions we have undertaken a taxon-wide search of the MAGUK family in eukaryotes. Our search included the complete genome sequence of the choanoflagellate *M. brevicollis *and the genome trace sequence data of *C. owczarzaki*, from which we have completed the full gene annotation by RACE PCR. Our data reveals that the MAGUK protein gene family already diverged in premetazoan lineages.

## Results

### Types of MAGUKs

Previous genomic comparisons have used different names to classify the distinct MAGUK classes [2,10]. Our data reveals that there are at least ten different types of MAGUKs. In the interest of clarity we have classified the MAGUK into 10 classes: MAGI, CACNB, ZO, CARMA, DLG5, DLG1-4, MPP1, MPP2-7, MPP5, and CASK. Those MAGUK classes with their corresponding protein domain architecture are shown in Figure 1. Although the protein domain architectures of MAGUKs are well conserved among the taxa within each class, some proteins have lost some of their domains, or their sequences are highly divergent. Figure 1 shows the canonical protein domain architecture; additional details and particularities are shown in Additional file 1.

### Phylogenetic analyses of the Guanylate Kinase domain

**Figure 2:**
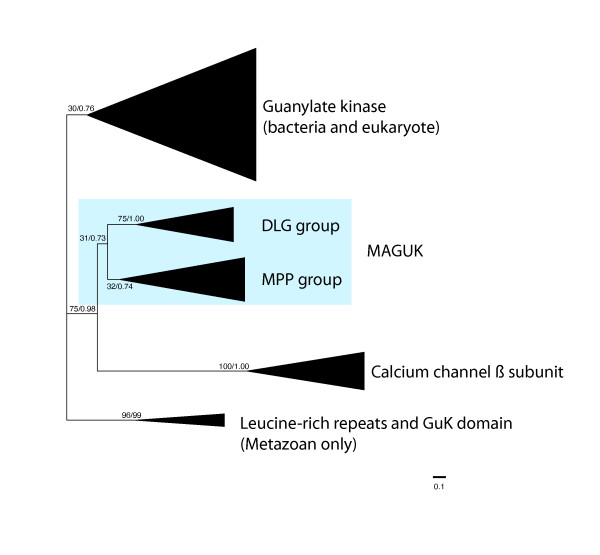
**Unrooted phylogenetic tree of GUK domain sequences**. The topology and branch lengths were obtained by maximum likelihood analysis performed in raxml. The schematic triangles length has been calculated to represent the average branch length of the tree. Statistical support obtained by 500-bootstrap raxml replicates, and bayesian posterior probability is shown for the main clades. The tree with all the taxa is shown in the Additional file 2.

Broad phylogenetic sampling of the guanylate kinase (GUK) domain was performed in order to check the monophyly of MAGUK within the larger GUK super-family. Although MAGI and CACNB homologs are considered to be members of MAGUK, their GUK domains are very divergent and the alignment quality decreases considerably when both of them are included. Thus, phylogenetic trees have been inferred either with MAGI or with CACNB representatives. The Maximum Likelihood (ML) phylogenetic tree that includes CACNB homologs is shown in Figure 2 (see Additional file 2 for the complete tree), while the analysis including MAGI is shown in Additional file 3. Statistical support for this last tree is very low, as it has a truncated GUK domain that leaves very few amino acid positions left for phylogenetic analysis. In any case, both topologies suggest that the "core MAGUKs" group (which comprises all MAGUK classes except for the MAGI and the CACNB classes) is monophyletic (75% ML bootstrap support). The trees also suggest that either the CACNB or the MAGI group is the sister-group of the "core MAGUKs". Which of those classes, MAGI or CACNB, is more closely related to the "core MAGUK" group remains elusive. In fact, whether any of them should really be considered MAGUKs is unclear even though MAGI and CACNB share the PDZ domain and the SH3 domain with the rest of MAGUKs respectively (see Figure 1). Their domain architecture may as well be a product of convergence.

### Survey of MAGUKs and phylogenetic analyses

**Figure 3:**
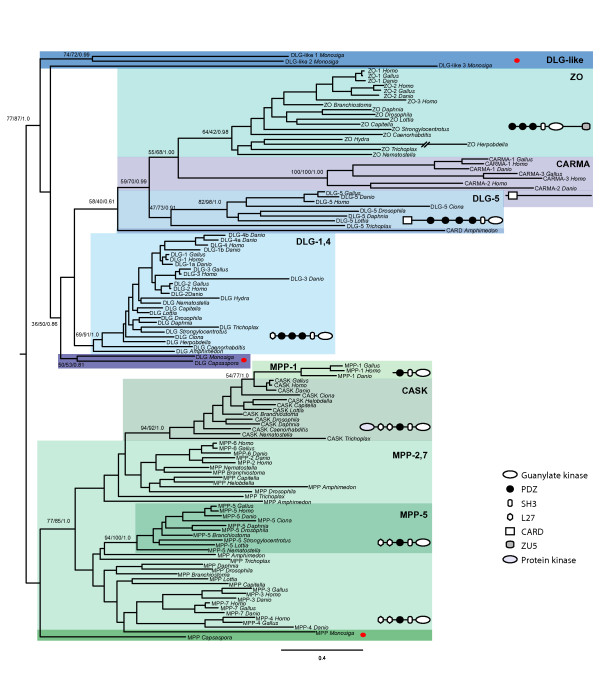
**Phylogeny of SH3+GUK domain sequences**. The topology and branch lengths were obtained by maximum likelihood analysis performed in raxml. The tree is rooted in the branch between the MPP and the DLG+DLG-like clades. Statistical support obtained by 100-bootstrap raxml replicates, 100-bootstrap phyml replicates, and bayesian posterior probability is shown for the main clades. The canonical domain architecture is shown for each MAGUK class.

Our survey and protein domain analysis of MAGUKs show that they are only present in Metazoa, choanoflagellates and the protist *C. owczarzaki*. We did not find any MAGUKs in any of the available fungi, amoebozoan, and other eukaryotic genomes. Eumetazoan (i.e. Cnidaria and Bilateria) taxa have at least one homolog representative of seven of the ten main classes of MAGUK, namely MAGI, CACNB, DLG 1-4, ZO, MPP2-7, MPP5, and CASK (see Figure 3). CARMA and MPP1 groups appear to be exclusive to Vertebrata. The placozoan *Trichoplax adhaerens *has a similar MAGUK repertoire as Cnidaria, as it only lacks the MPP5 class and it has instead a DLG5 homolog, which is actually missing in Cnidaria. However, the poriferan *Amphimedon queenslandica*, lacks homologs for MPP5, ZO, and CASK and some of its proteins branch in unclear positions within the tree.

An ML tree was inferred from the SH3 + GUK domains of the "core MAGUK" proteins. The MAGI and the CACNB classes were not included in the analysis. MAGI were excluded as they do not have the SH3 domain and their GUK domain is truncated. Similarly, although CACNB have SH3 and GUK, their sequences are very divergent and leave few amino acid left for phylogenetic analyses. We searched, among other eukaryotes, the genome sequences of *M. brevicollis*, *C. owczarzaki*, the cnidarian *Nematostella vectensi*s, the poriferan *A. queenslandica*, the placozoan *T. adhaerens*, plus a few representative bilaterians. When homologs from taxa with a relevant phylogenetic position, such as *Hydra magnipapillata*, were found, they were also included in the phylogenetic analysis.

The topology of the ML tree shows two statistically supported main clades (see Figure 3). One clade, which we name as the "DLG super class", consists of all DLGs plus ZO and CARMA. The other, which we name as the "MPP super class", comprises all MPPs and CASK. The different MAGUK classes are supported in our phylogenetic tree. Thus, ZO, CARMA, DLG5, DLG1-4, MPP5, MPP1, and CASK all appear as monophyletic groups, with varying bootstrap support. MPP 2-7 is the only group that appears as a paraphyletic group.

Within the DLG super class, ZO and CARMA group together and they branch as a sister-group to DLG5. An *A. queenslandica *protein with a domain architecture that is typical of DLG5 (it has the CARD domain, see Additional file 1) appears as the sister-group to the DLG5+ZO+CARMA clade. The DLGs of both *C. owczarzaki *and *M. brevicollis *group together as the sister-group to the whole DLG super class. Within the MPP super class, MPP1 groups within CASK, MPP5 forms a clear clade, while the remaining MPP genes do not appear to be monophyletic. *Capsaspora*-MPP branches as the sister-group of the entire MPP super class, whereas *Monosiga*-MPP appears within the metazoan MPP group.

Finally, three putative MAGUK-like genes from *M. brevicollis *branch in an intermediate position between the DLG and the MPP super classes.

To check the possibility than one or several lateral gene transfer (LGT) events may have occurred from metazoans to choanoflagellates and *C. owczarzaki*, we performed a neighbour-net analysis to see whether alternative trees may hint to a potential LGT event. The analysis clearly shows that the homologs of *C. owczarzaki *and *M. brevicollis *do not come from LGT from metazoans since they clearly group outside the major metazoan MAGUK types, as we would expect if LGT between metazoans and those protists had taken place (Additional file 4). Instead their homologs branch deep into the root of the tree.

### Protein domain architecture of MAGUKs in premetazoan taxa

**Figure 4:**
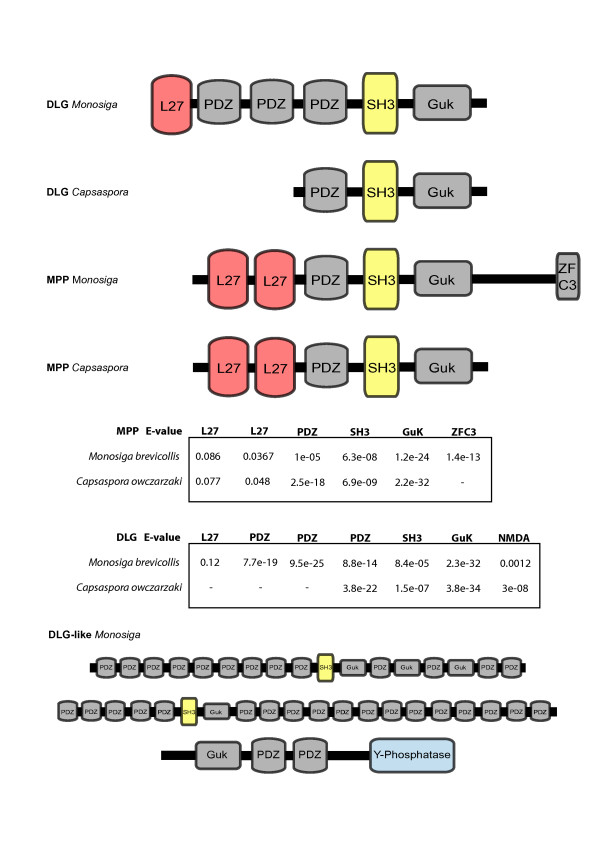
**Protein domain architectures for the Capsaspora and Monosiga MAGUK and MAGUK-like homologs, except for MAGI**. E-values as retrieved on PFAM are shown below for canonic MAGUK homologs protein domains.

Several MAGUKs are present in some non-metazoan taxa. Both the protist *C. owczarzaki *and the choanoflagellate *M. brevicollis *present putative MAGI, DLG and MPP homologs. The DLG and MPP proteins of *C. oczarzaki *and *M. brevicolli*s cluster basal to the DLG and MPP super classes respectively (except for the Monosiga-MPP, see Figure 3; Additional File 5 contains the taxa data). *M. brevicollis *has three additional MAGUK-like proteins, which branch in intermediate positions between the DLG and MPP super classes. The protein domain organization of *M. brevicollis *and *C. owczarzaki *MAGUKs are shown in Figure 4. The putative *Monosiga*-DLG, which branches as a sister-group to the *Capsaspora*-DLG, has the canonical DLG protein domain architecture, with an L27 domain, the three PDZ domains and an SH3 and a GUK domain. On the other hand *Capsaspora*-DLG lacks the N-terminal L27 domain and the first two PDZ domains (Figure 4). With regard to the MPP homologs, both *M. brevicollis *and *C. owczarzaki *present the standard MPP protein domain architecture: two L27 domains, one PDZ, and the SH3 and GUK domains. We found that the *Monosiga*-MPP has an additional C4 Zinc Finger domain (C3HC4) in the C-terminal end. The C3HC4 domain is a ring finger that plays a key role in the ubiquitination pathway of Metazoa. Interestingly, the DLG1 of vertebrates, which are also known as PSD-95 [2], also has an N-terminal PEST domain between L27 and the first PDZ domain, which is also involved in polyubiquitination [13].

The *Capsaspora*-MAGI has the canonical protein domain architecture of the MAGI class at the N-terminal end, with the PDZ, the GUK and the two WW domains. However, it lacks the PDZ domains at the C-terminal end as previously shown in [12]. Two putative MAGI homologs from *M. brevicollis *(*Monosiga*-MAGI-like) were identified in the GUK phylogenetic analysis (Additional file 3). They present diverging protein domain architectures, lacking the two WW domains seen in the typical MAGI proteins. Moreover, we have also identified three MAGUK-like proteins in *M. brevicollis *with unique protein domain architectures, in which the core SH3 and GUK domains are wrapped around several consecutive PDZ domains both at the N- and C-terminal ends (see Figure 4).

## Discussion

### Reconstruction of MAGUK diversity in the metazoan ancestor

**Figure 5:**
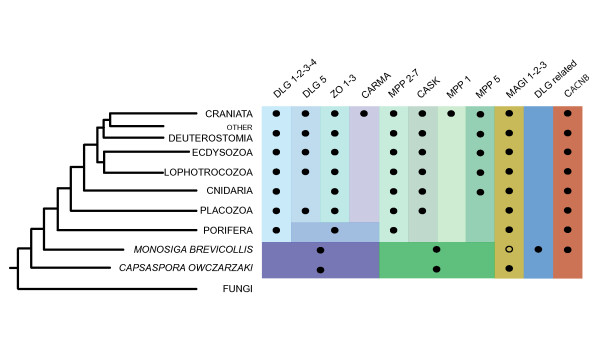
**Distribution of MAGUKs in opisthokonts**. Black circles means presence, whereas white circles means the homology assignment is not clear. The DLG and MPP columns indicate presence within that major clade (the super class) but without clear assignment to any of the inclusive classes (see text for further explanations).

Our survey of MAGUK proteins shows that three canonical MAGUKs are present in both the protist *C. owczarzaki *and the choanoflagellate *M. brevicollis *(Figure 5). Both organisms have homologs of the metazoan DLG and MPP super classes. Additionally, *C. owczarzaki *has a MAGI homolog as previously stated [12], whereas *M. brevicollis *has two putative MAGI-like homologs, although with divergent and unique protein domain architecture (Additional file 1). Since *C. owczarzaki *is most likely the sister-group of choanoflagellates and Metazoa [12,14-16], our results suggest that the common ancestor of Metazoa, *C. owczarzaki*, and choanoflagellates already had three types of MAGUK: a DLG-like, an MPP-like and a MAGI-like protein (Figure 5). A canonical MAGI was, thus, either lost or drastically diverged in the choanoflagellate lineage. Alternatively, the *Capsaspora*-MAGI may represent an independent acquisition of the PDZ-GUK-WW-WW domain architecture. Additional genomic data from other choanoflagellates will be needed to draw definitive conclusions. Although in theory one can not rule out the possibility than one or several lateral gene transfer (LGT) events may have occurred from metazoans to choanoflagellates and *C. owczarzaki*, we favour the hypothesis that the MAGUK protein family appeared prior to the divergence between *C. owczarzaki *and choanoflagellates. In fact, the neighbour-net analysis shows that the homologs of *C. owczarzaki *and *M. brevicollis *do not have a clear relationship with any of the metazoan MAGUK types, as we would expect if LGT between metazoans and those protists had taken place (see Additional file 4).

Interestingly, we have also identified three unique MAGUK-like proteins in *M. brevicollis*. Two of them present the canonical PDZ-SH3-GUK domains of MAGUK, but with several consecutive PDZ domains at both the N-terminal and C-terminal ends. One of those *M. brevicollis *MAGUK proteins homolog has several GUK duplications, and the third one has an additional protein domain: a phosphatase kinase (see Figure 4). All these MAGUK-like proteins constitute novel domain architectures that had not been found in any other organism up to now. Novel protein domain arrangements that include several consecutive copies of a domain have already been found in *M. brevicollis *[17]. Whether these MAGUK-like protein domain arrangements are also present in other choanoflagellates or in other opisthokont protists remains unclear. In any case, our data shows that choanoflagellates, or at least *M. brevicollis*, underwent an independent lineage-specific diversification of the MAGUK protein gene family. Functional analysis on these genes may clarify what roles the different genes are playing in *M. brevicollis*.

In fact, the role of MAGUK proteins in these premetazoan taxa remains an open question. In the sponge *A. queenslandica *both the DLG and MAGI homologs are specifically expressed only in epithelia-like tissue [18]. Moreover, it has been shown that *A. queenslandica *has and expresses several other components of the synaptic junction [18]. Interestingly, those components are expressed in the flask cells and the epidermis. It is therefore possible that the interaction between MAGUKs and the complex protein scaffolds found in eumetazoan post-synaptic scaffolds or in tight-junctions, was already present in sponge. To which components *M. brevicollis *or *C. owczarzaki *MAGUKs interact is unknown. Answering this is beyond the scope of this manuscript. Such information, however, may be crucial to decipher the role of MAGUKs in unicellular taxa. Moreover, additional data from colonial choanoflagellates or ichthyosporeans should yield important insights into this question, since they may harbor some scaffolding assemblages that enable the different cells in the colony to communicate.

### Evolutionary history of MAGUK

Our data show that although the MAGUK family most probably originated within choanozoans (i.e. unicellular lineages that are most closely related to Metazoa), it clearly expanded within metazoans. In fact, it seems that the expansion of MAGUKs in metazoans occurred at least in two distinctive periods in the course of evolution, as observed in many other protein families [19]. The first expansion occurred at the very early metazoan evolution before the divergence between sponges (or placozoans) and the rest of metazoans. The second expansion is likely to have happened at the early evolution of vertebrates. Accordingly, both Porifera and Placozoa have several extra MAGUKs classes, and Vertebrata has two exclusive classes, CARMA and MPP1, that were most probably generated by independent duplications in the vertebrate lineage.

The analysis of the protein domain organization mapped onto our phylogenetic analysis sheds some light on the evolutionary history of MAGUKs. For example, a duplication of a protein kinase domain followed by its recruitment in the N-terminal site of one MPP protein brought about a new type of MAGUK, the CASK class. This event probably took place either in the common ancestor of Placozoa and Eumetazoa, or at the origin of Metazoa followed by the loss of this MAGUK type in Porifera (Figure 5). Moreover, the vertebrate-specific MPP1 class seems to have derived from CASK, most probably by an additional duplication of the CASK homolog in vertebrates followed by the loss of the protein kinase domain and the two L27 domains. Vertebrates have the largest repertoire of MAGUKs. Not only do they have the vertebrate-specific MAGUK classes such as CARMA and MPP1, but they also have several genes that appear to have diverged in the early vertebrate evolution (e.g. in the DLG1-4, MAGI and ZO classes, vertebrates have three or four such genes). Although the sponge *A. queenslandica *has a MAGUK with the typical CARMA domain architecture (a CARD domain, a PDZ domain, an SH3 domain and a GUK domain), it branches as the sister-group to the entire DLG5, CARMA and ZO group. We hypothesize this *A. queenslandica *gene represents the ancestral form of this whole group. In fact, DLG5 and ZO are already present both in the placozoan *T. adhaerens *and in all other eumetazoans, except for the cnidarian *N. vectensis *which does not have a DLG5 homolog. The class CASK is also present both in *T. adhaerens *and in other eumetazoans. This indicates that CASK, DLG5 and ZO arose prior to the divergence between placozoans and the rest of eumetazoans.

It is worth mentioning that the interpretation of these results needs additional data, since the phylogenetic position of the basal metazoans is still under debate (see [20-24]). If the placozoan *T. adhaerens *is indeed the sister-group of cnidarians and Bilateria [20,24], then CASK, DLG5, and ZO appeared in the common ancestor of both placozoans and eumetazoans; and the DLG5 homolog was lost in the cnidarian lineage. However, if placozoans are indeed the most basal metazoans or sister-group to sponges [21,22], then the lineage leading to sponges (or *A. queenslandica*) did lose their ZO, CASK and DLG5 representatives. Finally, and even though the topology of the MAGUK tree remain the same with whatever method used, some of the nodes of our tree do not have high bootstrap values. Thus, some of the implications regarding the evolutionary history of MAGUK within metazoans may need to be revisited with additional data.

## Conclusions

In this study we have identified several MAGUK and MAGUK-like homologs in premetazoan taxa. Overall, our data show that the MAGUK protein gene family is not exclusive to Metazoa. This gene family most probably diversified within the opisthokonts before the divergence between *Capsaspora *and the Choanoflagellata + Metazoa clade. Thus, DLG, MPP and MAGI were already present in the last common ancestor of the Metazoa, choanoflagellates and *Capsaspora*+*Ministeria *(Filasterea) clade. The family further diversified within metazoans, most probably in two major episodes (early metazoan and early vertebrate evolution) and up to ten different MAGUK types evolved with different roles.

The choanoflagellate *M. brevicollis *has, independently of Metazoa, undergone a lineage-specific diversification of MAGUK-like proteins that have unique domain architectures. This represents a diversification independent from the one occurred in Metazoa. Additional genomic data from other choanoflagellate taxa will help elucidate whether this diversification is specific to choanoflagellates or just to the *M. brevicollis *species. Moreover, *M. brevicollis *has different and unique MAGI-like homologs, not found in Metazoa. Functional analyses will be needed to better understand the roles of MAGUKs in the unicellular relatives of Metazoa.

## Methods

### Database searching

All potential MAGUK sequences were obtained by performing blast searches (blastp, tblastn and psi-blast) against the Protein, Genome, and EST databases at the NCBI (National Center for Biotechnology Information) and against completed or on-going genome projects database at the JGI (Joint Genome Institute) and the Broad Institute. The amino acid sequences of the Homo sapiens orthologs were used as a query. The sequences retrieved that were not annotated as MAGUK were then blasted against NCBI CDD (Conserved Domain Database). Only those that retrieved a MAGUK protein domain architecture (a PDZ, SH3, GUK) were considered positives. Complete protein domain architectures were inferred by searching the PFAM and SMART databases with the complete sequences. Moreover, the putative positives were individually incorporated into a basic alignment of annotated sequences. Only those sequences that could unambiguously be aligned were used in the phylogenetic analysis.

In order to study the early evolution of the guanylate kinase domain, we performed additional phylogenetic analyses using sequences from a broad range of taxa including the Eubacteria. The same databases as above, together with the Genbank amino acid database, were searched with the HMMER3.0a2 program. In this case, the genes that did not show the typical MAGUK domain architecture were also included in the analyses. As the analyses were focused on the divergence between distinct subfamilies of the guanylate kinase family, the genes comprising each subfamily were properly removed in order to reduce the complexity of the tree.

### Phylogenetic analyses

Alignments of the SH3 + GUK domains were constructed using the Muscle [25] plug-in of the Geneious software (Biomatters Ltd, Auckland, New Zealand), before they were manually inspected and edited. Only those positions that were unambiguously aligned were included in the final analysis, which resulted in a total of 272 amino acid positions respectively. The final protein alignments can be downloaded from the webpage http://www.multicellgenome.com.

Maximum likelihood phylogenetic trees were estimated by RAxML [26] using a PROTGAMMAWAG model of evolution and with a gamma distribution (8 categories) (WAG+Γ). Statistical support was obtained from 100 bootstrap replicates using the Phyml program [27] following a LG+Γ+I model of evolution [28] with 4 rate categories, and from 100-bootstrap replicates in RAxML, using the PROTGAMMAWAG model of evolution and with a gamma distribution (4 categories). Bayesian trees were estimated on the MrBayes plug-in available on Geneious software. We ran four different Monte Carlo Markov chains using a WAG+Γ model of evolution with 4 rate categories. A total of 1,1 million generations were calculated with trees sampled every 200 generations and with a burn-in of 110,000.

The alignment of the sole guanylate kinase domain sequences from diverse taxa, including the Eubacteria, was generated manually as the sequences are divergent. The phylogenetic tree was inferred by RAxML with 500 bootstrap replicates. We inferred two phylogenetic trees with different repertoires of subfamilies, as the inclusion of both the MAGI and CACNB subfamilies in a single dataset results in a short alignment, that is insufficient for reliable tree inference. Both alignments can be downloaded from our webpage.

### Amplification of Capsaspora MAGUKs and annotation of Monosiga MAGUKs

*Capsaspora owczarzaki *data were obtained from genome sequence scaffolds from NCBI and the proteins were predicted both with AUGUSTUS [29] and GENSCAN [30]. Incomplete predictions were checked by rapid amplification of cDNA ends (RACE). Purified polyA+ messenger RNA and cDNA from *C. owczarzaki *were obtained as in [31]. The full sequence of the 5' and 3' ends of *C. owczarzaki *DLG and MPP were obtained by RACE, using a nested PCR and with primers designed from the original genome trace data. Primers used were as follows: CaDLG-3'RACE-1: 5' GACATGAAGGAGGAAAAGTTTATGG 3'; CaDLG-3'RACE-2: 5' ACAAAGGTGTTTGACACTGCAC 3'; CaDLG-5'RACE-1: 5' GTCGTTGACCTTCAAAATTTCATC 3'; CaDLG-5'RACE-2: 5' GAATCTTTGACACGTAAATGTTGG 3'; CaMPP-3'RACE-1: 5' AAGCTCCAAGAAGTCGTCAAGG 3'; CaMPP-3'RACE-2: 5' CAACGTTGGCTTCTTCTTTGAC 3'; CaMPP-5'RACE-1: 5' GTCTCGGTCAAAAAGTCCTTGAC 3'; CaMPP-5'RACE-2: 5' GTAAAGTCCTTGTTGGCGATCTT 3'. Sequences were obtained and analyzed as in [31]. These two sequences have been deposited at GenBank under the accession numbers GQ290472-GQ290473.

*Monosiga brevicollis *data were obtained from the predicted protein database. They were also confirmed by the AUGUSTUS and GENSCAN predictions from genome sequence scaffolds downloaded from JGI.

### Checking the network-like structure of MAGUK protein gene family

In order to check whether the ML tree follows a network-like or a tree-like structure, SplitsTree4 [32] was run to construct a neigbor-net using the alignment that includes the SH3 + GUK domains.

## Authors' contributions

AdM led on data collection, analysis, and data interpretation, contributed to writing. HS oversaw data collection, contributed to analysis and the writing. IR-T designed the experiments, oversaw the project and led on much of the writing. All authors read and approved the manuscript.

## Supplementary Material

Additional file 1 **Schematic MAGUK domain organization among Holozoa**. Domain structures of the key taxa are shown here to reveal the differential conservation in this gene family. Click here for file

Additional file 2 **Unrooted phylogenetic tree of the GUK domain sequences, including the Calcium channel B subunit**. The topology and branch lengths were obtained by maximum likelihood analysis performed in raxml. Statistical support was obtained by 500-bootstrap raxml replicates. Click here for file

Additional file 3 **Unrooted phylogenetic tree of the GUK domain sequences, including MAGI**. The topology and branch lengths were obtained by maximum likelihood analysis performed in raxml. Statistical support was obtained by 500-bootstrap raxml replicates. Click here for file

Additional file 4 **A neigbor-net of MAGUKs**. A neigbor-net constructed from the MAGUK alignment that includes the SH3 + GUK domains. Major groupings and the homologs of *C. owczarzaki *and *M. brevicollis *are indicated. Click here for file

Additional file 5 Taxa used in the phylogenetic analysis of Figure 3. Click here for file
