# Supplementary figures and images for: Evolution of the MAGUK protein gene family in premetazoan lineages
Source: BMC Evol Biol. 2010 Apr 1;10:93. doi: 10.1186/1471-2148-10-93 (PMC2859873; doi:10.1186/1471-2148-10-93)

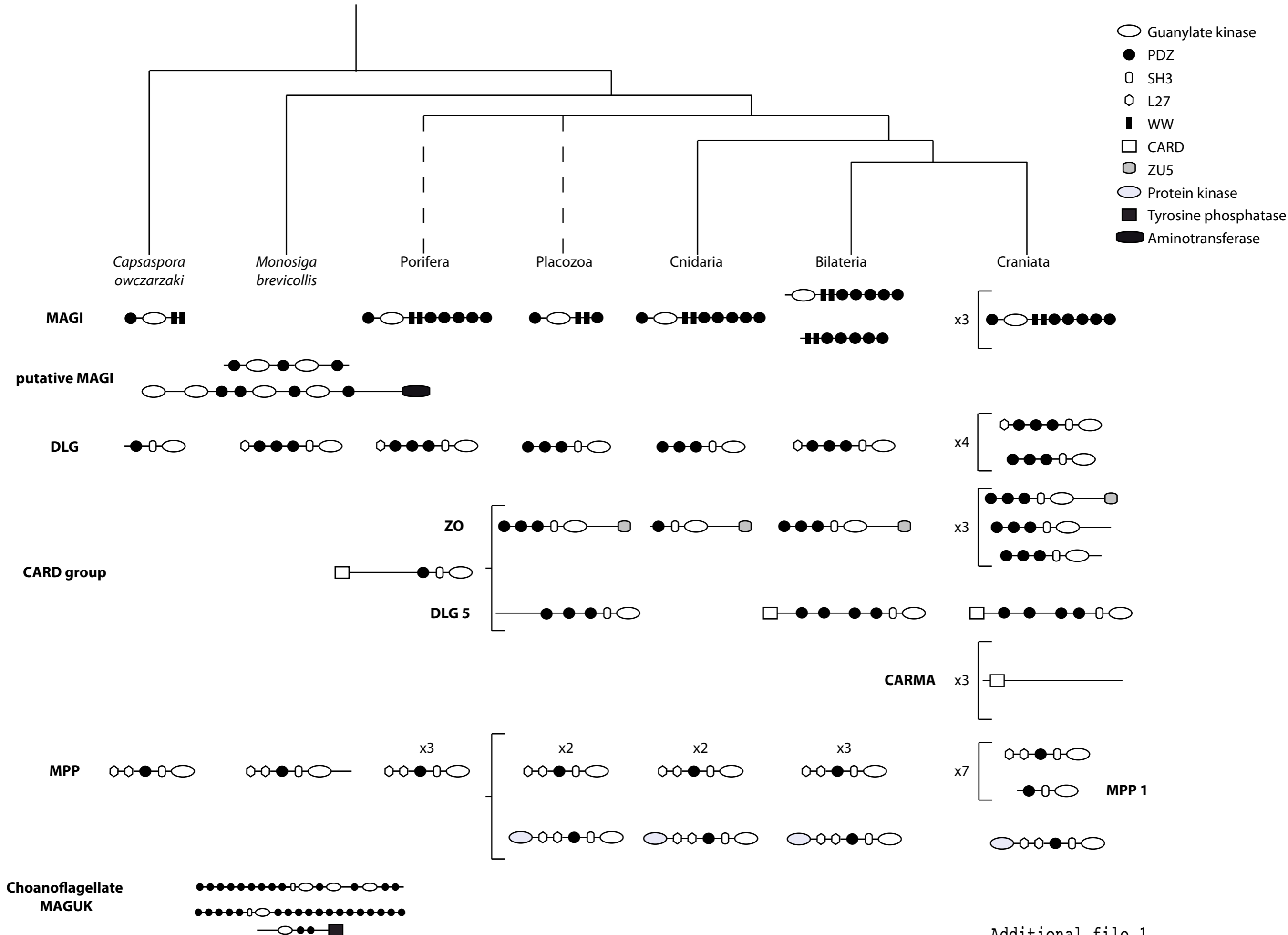

Supplement: Additional file 1 — Schematic MAGUK domain organization among Holozoa. Domain structures of the key taxa are shown here to reveal the differential conservation in this gene family. [file 1471-2148-10-93-S1.PDF]

—  
0.1

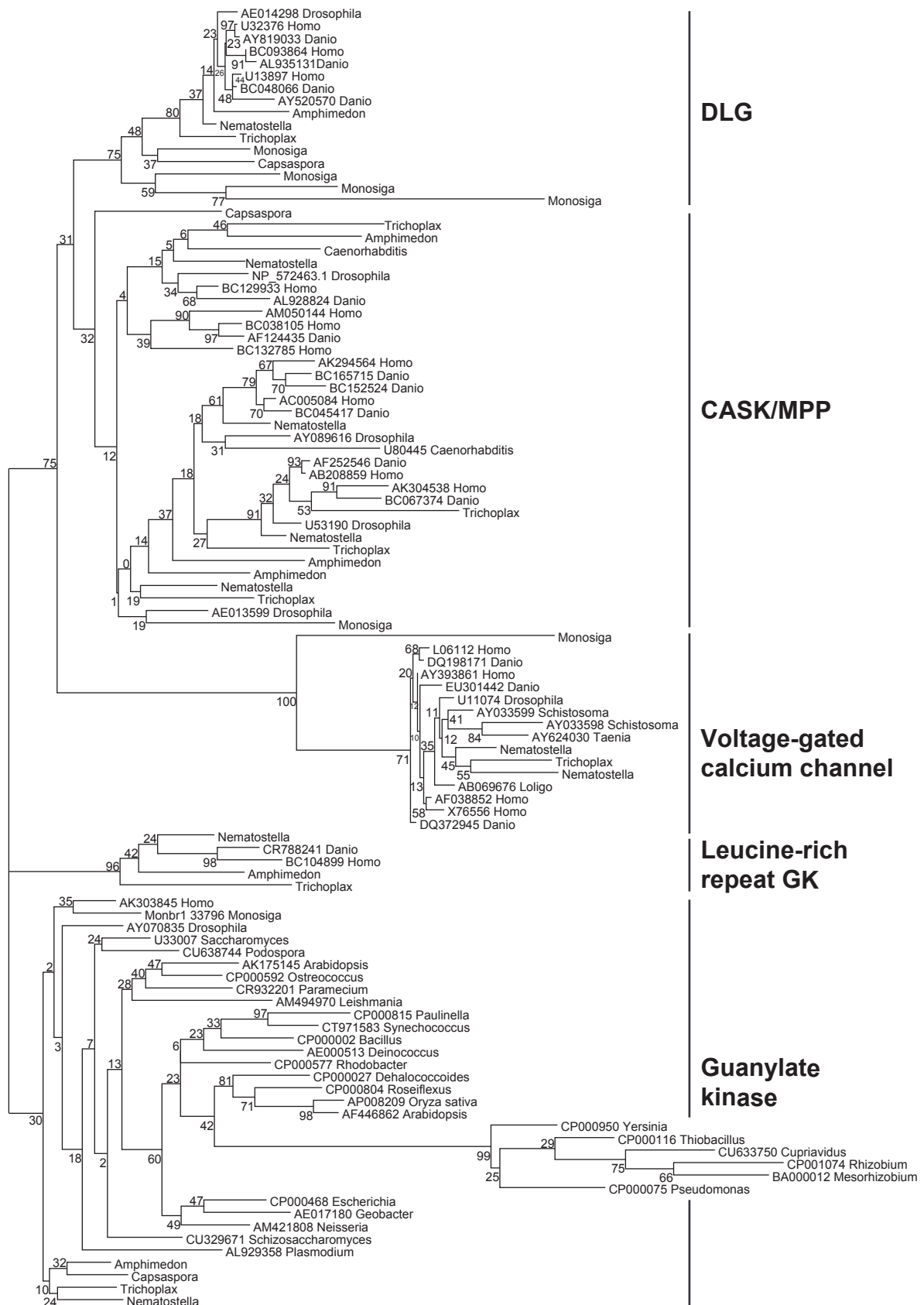

Supplement: Additional file 2 — Unrooted phylogenetic tree of the GUK domain sequences, including the Calcium channel B subunit. The topology and branch lengths were obtained by maximum likelihood analysis performed in raxml. Statistical support was obtained by 500-bootstrap raxml replicates. [file 1471-2148-10-93-S2.PDF]

0.1

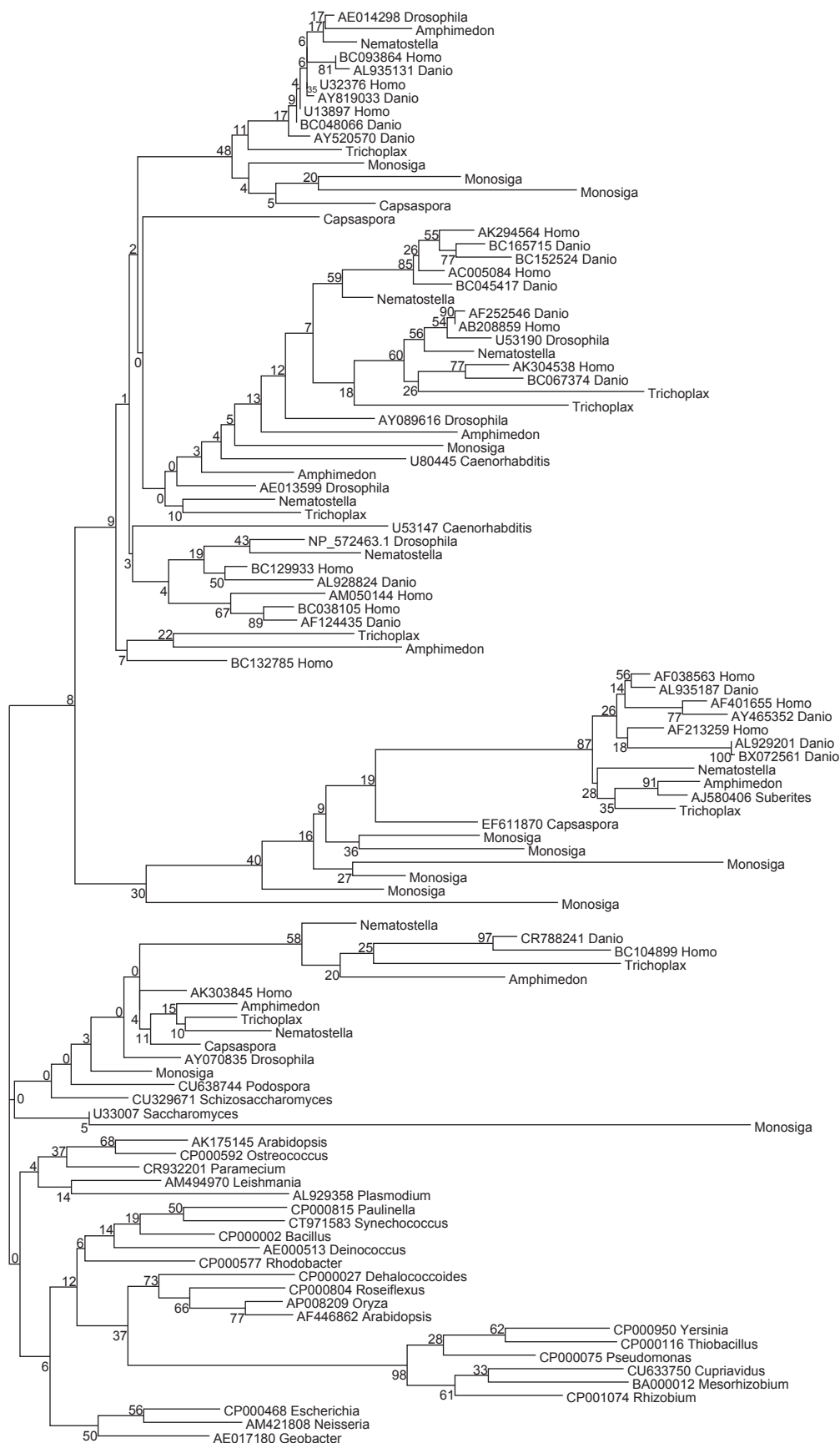

Supplement: Additional file 3 — Unrooted phylogenetic tree of the GUK domain sequences, including MAGI. The topology and branch lengths were obtained by maximum likelihood analysis performed in raxml. Statistical support was obtained by 500-bootstrap raxml replicates. [file 1471-2148-10-93-S3.PDF]

0.1

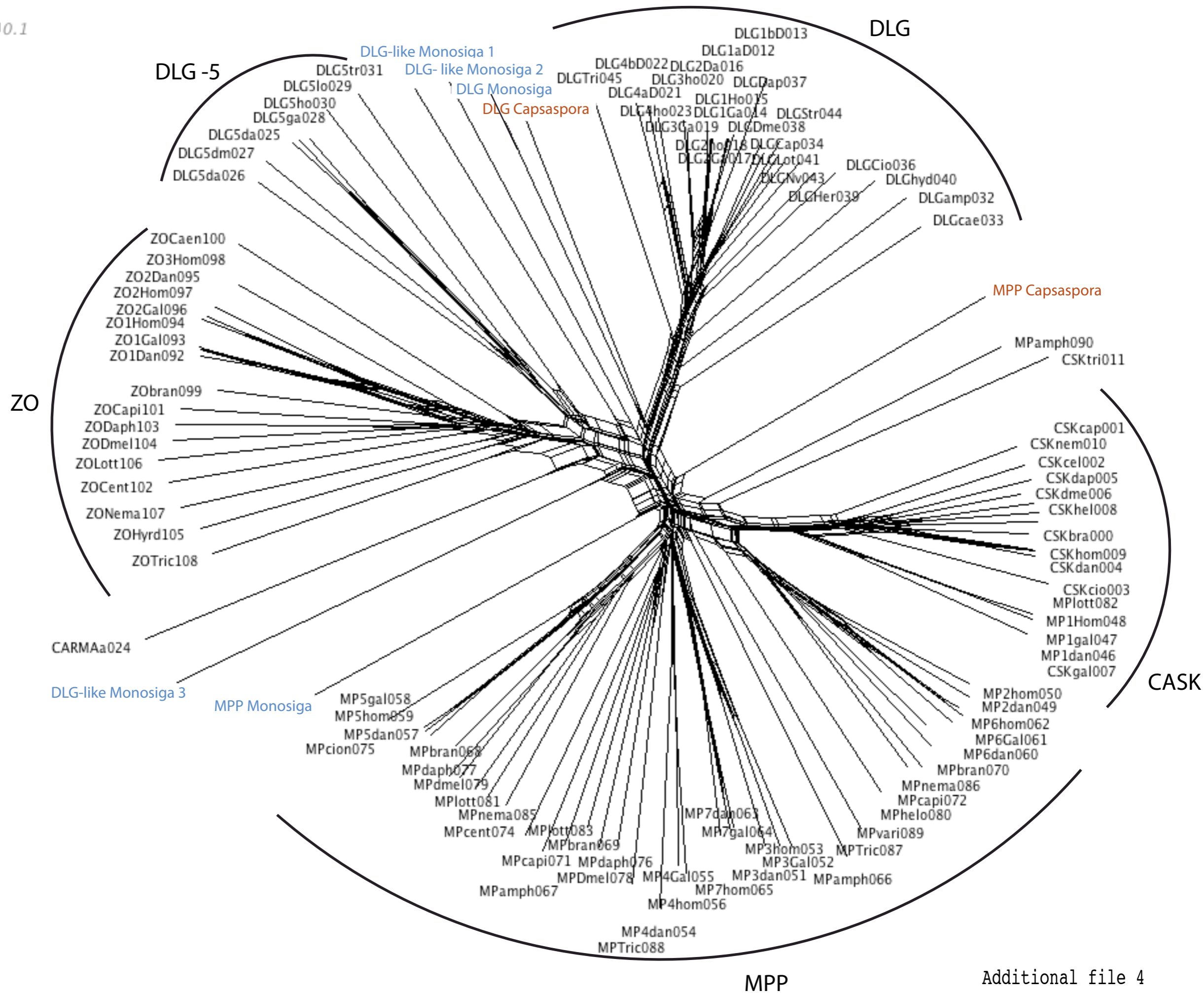

Supplement: Additional file 4 — A neigbor-net of MAGUKs. A neigbor-net constructed from the MAGUK alignment that includes the SH3 + GUK domains. Major groupings and the homologs of C. owczarzaki and M. brevicollis are indicated. [file 1471-2148-10-93-S4.PDF]
