# Supplementary material for: Evolution of the MAGUK protein gene family in premetazoan lineages
Source: BMC Evol Biol. 2010 Apr 1;10:93. doi: 10.1186/1471-2148-10-93 (PMC2859873; doi:10.1186/1471-2148-10-93)
Supplement: Additional file 5 — Taxa used in the phylogenetic analysis of Figure 3. [file 1471-2148-10-93-S5.PDF]

Gene list

CARD	Amphimedon queenslandica	[rspp] Aqu1.213008
CARMA 1	Danio rerio	XP_685144
CARMA 2	Danio rerio	XP_697127.3
CARMA 1	Gallus gallus	NP_001006161
CARMA 3	Gallus gallus	XP_416277.2
CARMA 1	Homo sapiens	NP_115791
CARMA 2	Homo sapiens	NP_077015
CARMA 3	Homo sapiens	NP_055365
CASK	Branchiostoma floridae	XP_002208829
CASK	Capitella capitata	jgi|Capca1|152517
CASK	Ciona intestinalis	jgi|Cioin2|285658
CASK	Caenorhabditis elegans	NP_001024587
CASK	Danio rerio	AAI62667.1
CASK	Daphnia pulex	jgi|Dappu1|195806
CASK	Drosophila melanogaster	NP_524441.2
CASK	Gallus gallus	XP_416769.2
CASK	Helobdella robusta	jgi|Helro1|87901
CASK	Homo sapiens	NP_003679.2
CASK	Lottia gigantea	jgi|Lotgi1|162383
CASK	Nematostella vectensis	XP_001631427.1
CASK	Trichoplax adhaerens	XP_002111823.1
DLG 1	Danio rerio	NP_001012388.1
DLG 1	Danio rerio	NP_955820.1
DLG 1	Gallus gallus	XP_422701.1
DLG 1	Homo sapiens	NP_001091894.1
DLG 2	Danio rerio	NP_001012378.1
DLG 2	Gallus gallus	XP_417217.2
DLG 2	Homo sapiens	EAW75095.1
DLG 3	Danio rerio	XP_001922578.1
DLG 3	Gallus gallus	XP_426264.2
DLG 3	Homo sapiens	NP_066943.2
DLG 4	Danio rerio	XP_001340947.2
DLG 4	Danio rerio	NP_999893.1
DLG 4	Homo sapiens	NP_001122299.1
DLG	Caenorhabditis elegans	Q18165_CAEEL
DLG	Capitella capitata	jgi|Capca1|217798
DLG	Capsaspora owczarzaki	GQ290473
DLG	Ciona savignyi	ensembl|ENSCSAVP0000001568
DLG	Daphnia pulex	jgi|Dappu1|214226
DLG	Drosophila melanogaster	NP_996406.1
DLG	Helobdella robusta	jgi|Helro1|75327
DLG	Hydra magnipapillata	XP_002168436.1
DLG	Lottia gigantea	jgi|Lotgi1|61304
DLG	Monosiga brevicollis	XP_001743865.1
DLG	Nematostella vectensis	jgi|Nemve1|161775|
DLG	Strongylocentrotus purpuratus	XP_001190785.1
DLG	Trichoplax adhaerens	XP_002108800.1
DLG 5	Ciona intestinalis	jgi|Cioin2|289933
DLG 5	Danio rerio	NP_001139075.1
DLG 5	Daphnia pulex	jgi|Dappu1|224336
DLG 5	Drosophila melanogaster	NP_609505.1
DLG 5	Gallus gallus	XP_421604.2
DLG 5	Homo sapiens	NP_004738.3
DLG 5	Lottia gigantea	jgi|Lotgi1|170600|
DLG 5	Trichoplax adhaerens	XP_002112079.1
MPP 1	Danio rerio	CAN87775.1
MPP 1	Gallus gallus	NP_001007918.1
MPP 1	Homo sapiens	NP_002427.1
MPP 2	Danio rerio	NP_001002223.1
MPP 2	Homo sapiens	NP_005365.3
MPP 6	Danio rerio	NP_001038242.1
MPP 6	Gallus gallus	XP_418721.2
MPP 6	Homo sapiens	NP_057531.2
MPP 2-6 like	Amphimedon queenslandica	[rspp] Aqu1.217120
MPP 2-6 like	Branchiostoma floridae	jgi|Brafl1|265353
MPP 2-6 like	Capitella capitata	jgi|Capca1|181775
MPP 2-6 like	Drosophila melanogaster	NP_995733.2
MPP 2-6 like	Helobdella robusta	jgi|Helro1|110223
MPP 2-6 like	Nematostella vectensis	XP_001625677.1
MPP 3	Danio rerio	XP_700838.2
MPP 3	Gallus gallus	XP_418108.2
MPP 3	Homo sapiens	EAW51661.1
MPP 4	Danio rerio	XP_685257.3
MPP 4	Gallus gallus	XP_001233047.1
MPP 4	Homo sapiens	NP_149055.1
MPP 7	Danio rerio	sp|Q6P0D7.1
MPP 7	Gallus gallus	XP_418583.2
MPP 7	Homo sapiens	NP_775767.2
MPP	Amphimedon queenslandica	[rspp] Aqu1.217121
MPP	Amphimedon queenslandica	[rspp] Aqu1.225220
MPP	Branchiostoma floridae	jgi|Brafl1|79765
MPP	Capitella capitata	jgi|Capca1|155756
MPP	Capsaspora owczarzaki	GQ290472
MPP	Daphnia pulex	jgi|Dappu1|98778
MPP	Drosophila melanogaster	NP_610642.1
MPP	Lottia gigantea	jgi|Lotgi1|92907
MPP	Monosiga brevicollis	XP_001745620.1
MPP	Nematostella vectensis	XP_001629601.1
MPP	Trichoplax adhaerens	XP_002110426.1
MPP	Trichoplax adhaerens	XP_002110949.1
MPP 5	Branchiostoma floridae	jgi|Brafl1|60486
MPP 5	Ciona intestinalis	jgi|Cioin2|250637
MPP 5	Danio rerio	NP_919344.1
MPP 5	Daphnia pulex	jgi|Dappu1|207442
MPP 5	Drosophila melanogaster	NP_001033835.2
MPP 5	Gallus gallus	XP_421200.1
MPP 5	Homo sapiens	NP_071919.2
MPP 5	Lottia gigantea	jgi|Lotgi1|115808
MPP 5	Strongylocentrotus purpuratus	XP_784409.2
ZO 1	Danio rerio	XP_001922690.1
ZO 1	Gallus gallus	XP_413773.2
ZO 1	Homo sapiens	NP_783297.2
ZO 2	Danio rerio	XP_693818.3
ZO 2	Gallus gallus	NP_990249.1
ZO 2	Homo sapiens	AAD20387.2
ZO 3	Homo sapiens	AAI08908.2
ZO	Branchiostoma floridae	jgi|Brafl1|93411
ZO	Caenorhabditis elegans	NP_001021685.1
ZO	Capitella capitata	jgi|Capca1|221876
ZO	Daphnia pulex	jgi|Dappu1|227765
ZO	Drosophila melanogaster	BAA11923.1
ZO	Helobdella robusta	jgi|Helro1|176655
ZO	Hydra magnipapillata	AAK28322.1
ZO	Lottia gigantea	jgi|Lotgi1|164754
ZO	Nematostella vectensis	XP_001633912.1
ZO	Strongylocentrotus purpuratus	XP_782687.2
ZO	Trichoplax adhaerens	XP_002116319.1
DLG-like 1	Monosiga brevicollis	XP_001742640.1
DLG-like 2	Monosiga brevicollis	XP_001744982
DLG-like 3	Monosiga brevicollis	XP_001748617

Additional file 5
